# Identification of Hookworm DAF-16/FOXO Response Elements and Direct Gene Targets

**DOI:** 10.1371/journal.pone.0012289

**Published:** 2010-08-19

**Authors:** Xin Gao, Zhengyuan Wang, John Martin, Sahar Abubucker, Xu Zhang, Makedonka Mitreva, John M. Hawdon

**Affiliations:** 1 Department of Microbiology and Tropical Medicine, The George Washington University Medical Center, Washington, District of Columbia, United States of America; 2 The Genome Center, Department of Genetics, Washington University School of Medicine, St. Louis, Missouri, United States of America; New England Biolabs, United States of America

## Abstract

**Background:**

The infective stage of the parasitic nematode hookworm is developmentally arrested in the environment and needs to infect a specific host to complete its life cycle. The canine hookworm (*Ancylostoma caninum*) is an excellent model for investigating human hookworm infections. The transcription factor of *A. caninum*, *Ac*-DAF-16, which has a characteristic fork head or “winged helix” DNA binding domain (DBD), has been implicated in the resumption of hookworm development in the host. However, the precise roles of *Ac*-DAF-16 in hookworm parasitism and its downstream targets are unknown. In the present study, we combined molecular techniques and bioinformatics to identify a group of *Ac*-DAF-16 binding sites and target genes.

**Methodology/Principal Findings:**

The DNA binding domain of *Ac*-DAF-16 was used to select genomic fragments by *in vitro* genomic selection. Twenty four bound genomic fragments were analyzed for the presence of the DAF-16 family binding element (DBE) and possible alternative *Ac*-DAF-16 bind motifs. The 22 genes linked to these genomic fragments were identified using bioinformatics tools and defined as candidate direct gene targets of *Ac*-DAF-16. Their developmental stage-specific expression patterns were examined. Also, a new putative DAF-16 binding element was identified.

**Conclusions/Significance:**

Our results show that *Ac*-DAF-16 is involved in diverse biological processes throughout hookworm development. Further investigation of these target genes will provide insights into the molecular basis by which *Ac*-DAF-16 regulates its downstream gene network in hookworm infection.

## Introduction

Many parasitic nematodes, including hookworms, infect the definitive host as developmentally arrested third-stage larvae (L3). The L3 is analogous to the dauer stage of the free-living nematode *Caenorhabditis elegans* in many biological aspects [1], [2], [3]. The FOXO-family forkhead transcription factor DAF-16 mediates dauer formation of *C. elegans* in response to cues indicating poor environmental conditions. When conditions improve, DAF-16 is negatively regulated by an insulin-like signaling (ILS) pathway that culminates in transport of phosphorylated DAF-16 out of the nucleus [4], [5], [6], [7], [8], [9], [10]. The primary protein structure of DAF-16 contains a conserved forkhead or “winged helix” DNA binding domain (DBD) with three major α-helices and two large wing-like loops [11], [12], [13]. Orthologs of DAF-16 have been recently characterized in the parasitic nematodes *Ancylostoma caninum* (*Ac*-DAF-16), *Strongyloides stercoralis* (*Ss*-DAF-16), and *Haemonchus contortus* (*Hc*-DAF-16) [14], [15], [16], [17]. Heterologous rescue of *C. elegans daf-16* mutants [15], [16] and reporter assays in mammalian cells [17], [18] indicate that parasitic nematode DAF-16 orthologs play similar regulatory roles during development, providing further support for the use of dauer exit as a model to investigate the molecular events of infection and successful establishment of a parasitic relationship with the host [1].

Murine DAF-16/FOXO was shown to bind an 8-bp consensus DAF-16 family member binding element (DBE) *in vitro* [19]. Several approaches have since been used to identify DAF-16 target genes in *C. elegans*, with the results suggesting that DAF-16 is recruited to a large number of promoters to modulate the expression of genes involved in development, metabolism, stress responses, and longevity [20], [21], [22], [23].

Our lab has been focusing on the infectious process of hookworms, one of the most common infectious diseases in tropical and subtropical countries, causing anemia and malnutrition in almost a billion people [24]. The canine species *A. caninum* is a commonly used model for investigation of human hookworm infections. The DAF-16 ortholog from *A. caninum* (*Ac*-DAF-16) was shown to be transcriptionally active and capable of interacting with a hookworm 14-3-3 protein, suggesting a critical role in gene expression associated with hookworm L3 development and the transition to parasitism [17], [18]. Given the functional conservation between dauer recovery and hookworm infection, there is considerable interest in the transcriptional outputs of DAF-16 in hookworm and their function in parasitic development. Dissecting the hookworm DAF-16 downstream effector network will have important implications in the development of new intervention strategies for hookworm and other nematode infections.

The present study utilizes *in vitro* genomic selection, a technique built on the concept of systematic evolution of ligands by exponential enrichment (SELEX), where natural genomic sequences are used as a source for selection and amplification [25]. A combination of *in vitro* genomic selection and subsequent cloning has been developed as a powerful method to identify naturally occurring DNA-binding sites in a genomic context and provide a foundation for investigation of the *in vivo* targets of DNA-binding proteins [26], [27], [28]. We employed the *in vitro* genomic selection strategy using *Ac*-DAF-16 DBD to screen digested hookworm genomic DNA, and identified high-affinity binding sites in the hookworm genome and potential *Ac*-DAF-16 gene targets. Finally, the expression profile of the *Ac*-DAF-16 related transcripts was determined by examining cDNAs from four developmental stages of *A. caninum*.

## Results

### *Ac*-DAF-16 DNA binding domain (DBD) expression and purification

**Figure 1:**
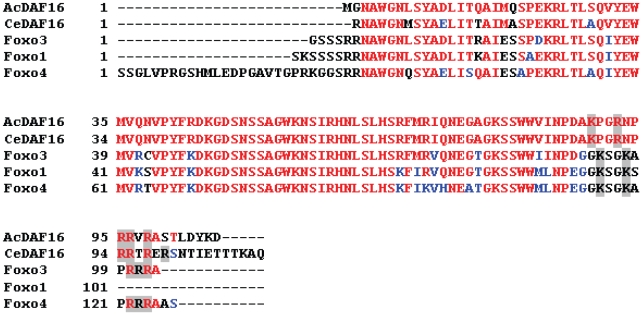
Amino acid sequences of DNA binding domains from selected FOXO transcription factors were aligned using CLUSTAL W software on the San Diego Supercomputer Center Biology Workbench server (http://workbench.SDSC.edu) and displayed using BOXSHADE3.21 software located on Swiss EMBnet server (http://www.ch.embnet.org). Identical amino acids are in red type, and conserved amino acids in blue. C-terminal arginine and lysine residues are shaded. The DBDs are from the following species: AcDAF16 (*Ancylostoma caninum* accession ACD85816); CeDAF16 (*Caenorhabditis elegans*, AAB84390); Foxo3, (*Mus musculus,* AAH19532); Foxo1 (*Homo sapiens,* AAH70065); Foxo4 (*Homo sapiens,* AAI06762).

Amino acid sequence alignment of different FOXO proteins revealed that the DBD is approximately 100 amino acids-long, with a conserved N-terminal region, and a divergent, but arginine/lysine-rich C-terminal region (Fig. 1). Several crystal structures for FOXO transcription factor DBDs have been solved, and reveal that the structural basis for FOXO protein recognition of DNA comes from direct base-specific contacts as well as phosphate contacts between DNA molecules and critical C-terminal arginine/lysine amino acid residues in the FOXO DBD [29], [30]. Based on the information from those crystal structures and sequence alignment between different DAF16/FOXO molecules, the DBD of *Ac-*DAF-16 was defined to start at amino acid Asn214 and end at amino acid Asp314.

**Figure 2:**
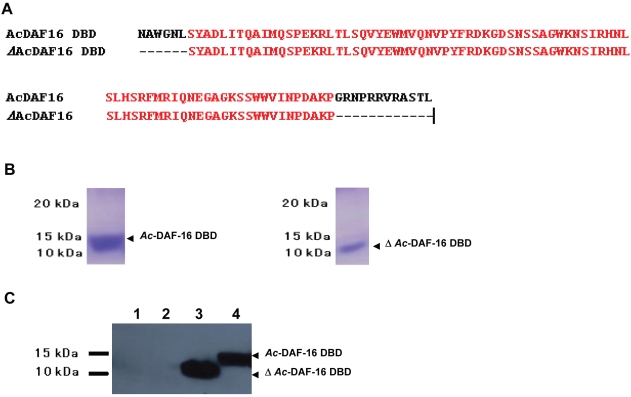
Expression of *Ac*-DAF-16 DBD (aa 214–314) and *Δ Ac*-DAF-16 DBD (aa 220–292) in *E. coli* Rosetta (DE3) cells. (A) Amino acid sequences of *Ac*-DAF-16 DBD and *Δ Ac*-DAF-16 DBD. Identical amino acids are in red. (B) Coomassie staining of purified r*Ac*-DAF-16 DBD and rΔ*Ac*-DAF-16 DBD. (C) Western blot of r*Ac*-DAF-16 DBD and r*ΔAc*-DAF-16 DBD probed with anti-his (C-term) antibody. Lane 1, non-transformed Rosetta (DE3) cells; Lane 2, pET28a-*Ac*-DAF-16 DBD transformed Rosetta (DE3) cells in the absence of IPTG; Lane 3, pET28a-*ΔAc*-DAF-16 DBD transformed Rosetta (DE3) cells induced with IPTG; Lane 4, pET28a-*Ac*-DAF-16 DBD transformed Rosetta (DE3) cells induced with IPTG. The arrowheads indicate the position of *Ac*-DAF-16 DBD or *Δ Ac*-DAF-16 DBD.

Properly functioning recombinant hookworm DAF-16 DBD peptide was required for the *in vitro* genomic selection technique. A fragment of 303 bp corresponding to *Ac*-DAF-16 DBD (aa 214-314), and a 249 bp fragment corresponding to a truncated *Ac*-DAF-16 DBD (aa 220–302) lacking the arginine/lysine-rich C-terminal region (*ΔAc*-DAF-16 DBD) were cloned and expressed (Fig. 2A). The calculated molecular masses for the coding regions of these two constructs are 14047.4 Da (pET28a-*Ac*-DAF-16 DBD) and 12510.6 Da (pET28a-Δ*Ac*-DAF-16 DBD). As expected, bands corresponding to the predicted molecular weights (14 kDa for r*Ac*-DAF-16 DBD, and 12 kDa for r*Δ Ac*-DAF-16 DBD) were detected by Coomassie Blue staining (Fig. 2B). Immunoblots using an anti-His tag (C-term) antibody indicated that the bands were present only in *E. coli* cultures that had been transformed with the expression constructs and induced with IPTG (Fig. 2C, Lanes 3 and 4).

**Figure 3:**
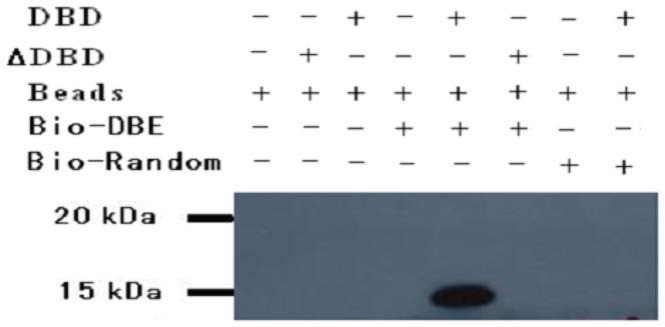
Streptavidin bead pull-down to detect DBE binding activity of recombinant *Ac*-DAF-16 DBD and Δ*Ac*-DAF-16 DBD. Biotinylated dsDBE (Bio-DBE) was incubated with purified *Ac*-DAF-16 DBD or Δ*Ac*-DAF-16 DBD, and peptide/oligonucleotides complexes were pulled down with streptavidin conjugated Sepharose beads. Precipitated DBD/oligo complexes were separated by SDS-PAGE and blotted to PVDF membranes for Western blotting using an anti-His (C-term) antibody. Bio-random represents a biotinylated oligomer of random sequence.

*Ac*-DAF-16 was previously shown to bind to the consensus DBE sequence and initiate reporter gene transcription [17]. Our pull-down assay results (Fig. 3) indicated that r*Ac*-DAF-16 DBD, but not r*Δ Ac*-DAF-16 DBD, recognizes and binds strongly to the conserved DBE, indicating that the arginine/lysine -rich section at the C-terminus of DBD is critical for its binding activity.

### *In vitro* genomic selection

**Figure 4:**
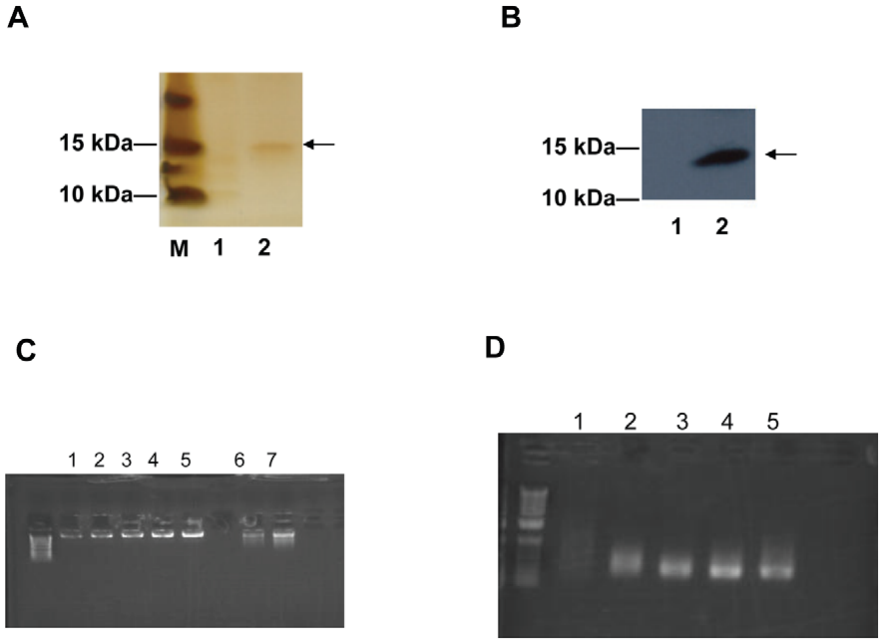
*In vitro* genomic selection of *A. caninum* DNA fragments containing *Ac*-DAF-16 binding sites. (A–B) Immobilized r*Ac*-DAF-16 DBD on Anti-FLAG M2 matrix confirmed by silver staining (A) and Western Blot with anti-FLAG antibody (B). The arrowheads indicate the position of *Ac*-DAF-16 DBD. M, protein standard; lane 1, Anti-FLAG M2 matrix; lane 2, anti-FLAG M2 matrix incubated with 2 ug of *Ac*-DAF-16 DBD and washed with 500 mM KCl. (C) *A. caninum* genomic DNA preparation. A 0.8% agarose gel was used to examine the DNA quality, and increasing amounts of a λ DNA standard were loaded to estimate DNA concentration. Lane 1–5, 30 ng λ DNA standard, 60 ng λ DNA standard, 90 ng λ DNA standard, 120 ng λ DNA standard, 150 ng λ DNA standard, respectively; lane 6, 2.5 µL of *A. caninum* genomic DNA sample; Lane7, 5 µL of *A. caninum* genomic DNA sample. (D) PCR amplification of the genomic fragments after each selection round. Lane 1, *A. caninum* genomic DNA sample cut with *Bfu*CI; lane 2, 1st round purified PCR product; lane 3, 2nd round purified PCR product; lane 4, 3rd round purified PCR product; lane 5, 4th round purified PCR product.

The *in vitro* genomic selection enabled unbiased identification of transcription factor binding sites in the absence of influence from chromatin and other cofactors [25]. Immobilized r*Ac*-DAF-16 DBD was first prepared by binding to anti-FLAG M2 affinity matrix, which was confirmed by silver staining (Fig. 4 A) and Western blot (Fig. 4 B), and used to screen *Bfu*CI digested *A. caninum* total genomic DNA (Fig. 4C). Discrete bands appeared progressively over the four rounds of binding and PCR amplification (Fig. 4D, lanes 2 to 5), suggesting preferential amplification of particular genomic fragments. Cloning the amplified DNA fragments from the fourth round resulted in a total of 311 clones (117 pBluescript KS+ constructs and 194 pGEM-T Easy constructs), and high-quality sequences were obtained for 274 of them. The length ranged from 100 bp to 300 bp. Sequence analysis showed that 198 sequences contained low-complexity microsatellite regions, which were characterized by the presence of the repetitive trinucleotide “GTT” or its reverse complement “AAC” with a repeat number of 5 to 15. The remaining 76 sequences represented 25 distinct genomic fragments, and 24 of them were successfully mapped to *A. caninum* genomic sequences. A single fragment remained unidentified after extensive sequence similarity searching against nucleotide databases at Genebank, suggesting that it might by located in a section of the *A. caninum* genome that has not been sequenced. Five of the 25 fragments were overrepresented, and the remaining 20 fragments were recovered once or twice (Table 1). The sequences of the distinct genomic fragments were submitted to the Genome Survey Sequence database (dbGSS) at the NCBI, and the accession numbers reported in Table S1.

**Table 1:** Proposed binding motifs found in fragments that bound to DAF-16 DBD *in vitro*.

Genomc selection clone #	Motif present	Occurrence Rate
Fragment 1.11	TTGTTTAC (N)_n_ TTGTTTAC	18
Fragment 1.25	CATTTGTT a	10
Fragment 1.3	GAAAACAA b (N)_n_ GTAAACAT b	1
Fragment 1.4	TTGTTTAT b	1
Fragment 2. 10	*GAGAAG*	1
Fragment 2.14	GTAAACAT b	1
Fragment 2.18	*GACAAG*	2
Fragment 2.19	GTAAACAA (N)_n_ GTAAATAA	1
Fragment 2.23	AACAAATA a,b	12
Fragment G2.28	AACAAATA a,b	1
Fragment G2.38	*GACATG* (N)_n_ *GAGAAG*	1
Fragment 3.23	*GACATG* (N)_n_ *GACATG*	1
Fragment 3.28	*GGCAAG* c	1
Fragment 3.30		7
Fragment G4.41	CTGTTTAC b (N)_n_ CTGTTTAC b	1
Fragment 4.26	ATAAACAA (N)_n_ GTAAATAA	7
Fragment 4.6	GATTTGTT a,b	2
Fragment 5.20	*GGCAAG* c	1
Fragment G5.21	*GACAAG* (N)_n_ *GAGAAG*	1
Fragment G5.7	*TACAAG* c (N)_n_ *GGCAAG* c	1
Fragment 6.33		1
Fragment 6.34	TTGTTTAC	1
Fragment 6.48		1
Fragment 8.1	TATTTGTT (N)_n_ CATTTGTT a,b	1

Predicted new r*Ac*-DAF-16 binding sites were indicated as italic.

a Reverse DBE.

b One mismatch compared with consensus DBE.

c One mismatch compared with predicted GAC/GAA/TG binding site.

A control selection was performed to control for non-specific binding to the antibody-bead matrix. A total of 211 clones were picked, and 190 high quality sequences were obtained. Sequence analysis showed that anti-FLAG M2 affinity matrix bound to a different set of hookworm genome fragments (Table S2). Among these sequences, only one was shared with the DAF-16 DBD-selected fragments, indicating that selection using the DBD generated specific genomic fragments associated with DAF-16 binding elements.

### Motif analysis of r*Ac*-DAF-16 DBD bound hookworm genome fragments

**Figure 5:**
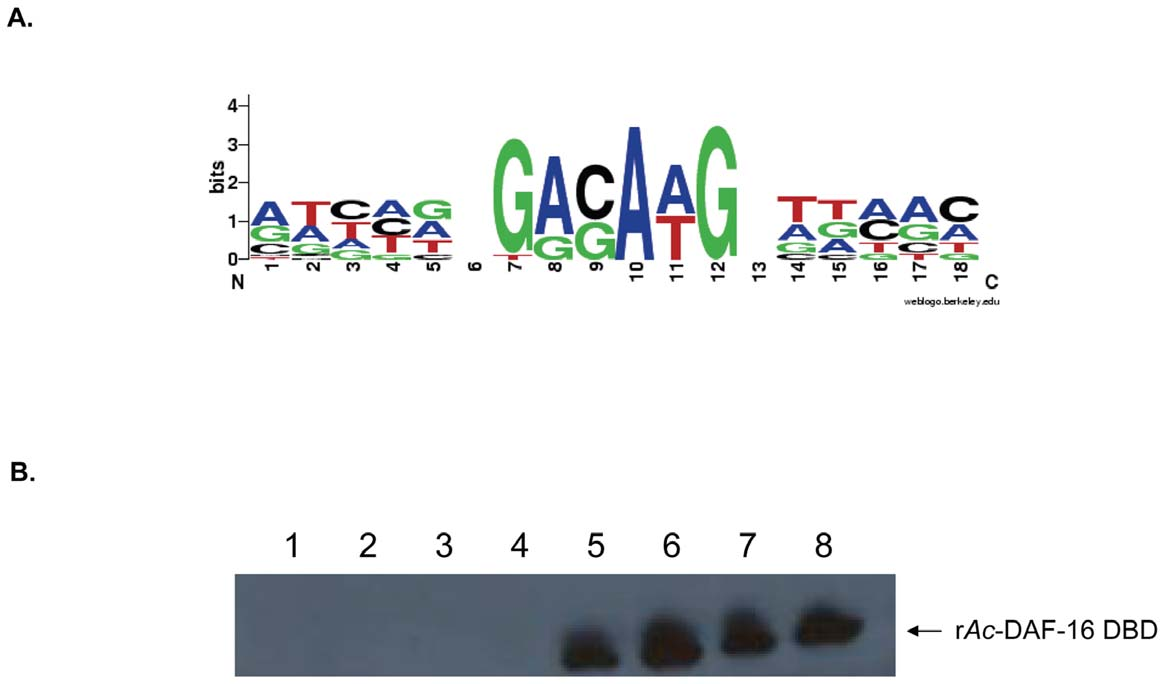
Identification of a novel DAF-16 DBD binding element. (A) Sequence logo of the putative DBD binding motif discovered using Gibbs Motif Sampler in bound fragments lacking a canonical DBE. (B) Streptavidin bead pull-down to detect the binding activity between positively selected genomic fragments and r*Ac*-DAF-16 DBD. Biotin labeled PCR products were incubated with r*Ac*-DAF-16 DBD. The protein/biotinylated PCR amplicon complexes were separated by SDS-PAGE and blotted to PVDF membrances for Western Blotting using anti-FLAG antibody. Lane1–4, Genome fragments from control selection; Lane 5, Fragment 3.23 (GACAAG motif); Lane 6, Fragment 3.28 (GACAAG motif); Lane 7, Fragment 4.6 (DBE); Lane 8, Fragment 2.23 (DBE).

The consensus DBE is an 8-bp oligonucleotide, with the sequence 5′-TTGTTTAC-3′ [19]. The core sequence of DBE is TGTT, and a single base-pair replacement in this sequence might significantly weaken its interaction with forkhead proteins. Therefore, only a single mismatch outside of the DBE core sequence was allowed when the 24 selectively bound fragments were inspected for DBEs. Using this criterion, 13 fragments contained the DBE or its reverse counterpart (Table 1), and two contained two copies of the DBE arranged as direct repeats separated by variable length of nucleotides. The remaining 11 genomic fragments were analyzed further, and a new over-represented sequence, 5′-GAC/GAA/TG-3′, was found, occurring 17 times in 9 of those fragments (Fig. 5a, Table 1). Genomic fragments selected by the anti-FLAG M2 control were also analyzed for over-represented motifs. One element (5′- AGGAAGAG- 3′) was found in 36% of the control fragments, but only 5% (5/103) of the fragments contained a DBE-like element (Table S2), indicating that the DBE and over-represented GAC/GAA/TG motif were bound specifically by r*Ac*-DAF-16 DBD.

The newly identified motif was tested for its ability to bind to *Ac*-DAF-16 DBD. Oligos containing the four most abundant motifs (5′-GACAAG-3′, 5′-GACATG-3′, 5′-GAGAAG-3′, 5′-GAGATG-3′) were tested in a streptavidin bead pull-down assay. The oligos failed to pull down r*Ac*-DAF-16 DBD (data not shown). One possible explanation for this result was that additional flanking sequence might be required for efficient binding. To test this possibility, biotinylated T7 primer was used to amplify representative cloned genomic fragments, which were tested for their ability to bind the DAF-16 DBD. As shown in Figure 5b, r*Ac*-DAF-16 DBD strongly bound to positively selected genomic fragments that contained a DBE (fragments 4.6 and 2.23) or the newly identified motif (fragments 3.23 and 3.28), but not to the anti-FLAG M2 affinity matrix selected (control) genomic fragments. This suggests that sequence flanking the element is required for in vitro binding, and that the newly identified motif specifically binds to the *Ac*-DAF-16 DBD when located in the proper context.

### Genes or gene clusters linked to the recovered r*Ac*-DAF-16 DBD bound fragments

Transcription factor binding sites are typically linked to their direct target genes. To identify the genes or gene clusters linked to the r*Ac*-DAF-16 DBD bound fragments, approximately 6 kb of the *A. caninum* genome scaffold sequences [39], (Mitreva, unpublished) containing the fragments at the center were searched against stage-specific hookworm cDNA databases. Nineteen of the 24 genome scaffolds retrieved about 5000 cDNAs from different developmental stages, one scaffold matched an rRNA region, and the other four scaffolds either had poor sequence alignments or failed to retrieve any cDNAs (Table 2, Table S3).

**Table 2:** Expression pattern of genes that bound to DAF-16 DBD in vitro.

Genomic Fragment	Contig ID^1^	Homology/description	Adult Expression Pattern^2^	Larval Expression Pattern^3^
1.3	11016	cAMP-dependent protein kinase (PKA)	down in F	up in aL3
3.28	00807			larval specific; up in aL3
1.4	52455		nc	nc
4.6	25170	Hypothetical protein R05C11.4		larval specific; up in aL3
6.34	03743	ABC transporter, class F family (abcf-2)	up in F	up in aL3
1.25	12656		M specific	up in aL3
2.18	20406	Neuro-peptide like protein family (NLP-2)		larval specific
2.18	40879	Hypothetical protein T01B6.1	F specific	down in aL3
2.19	09925	cuticlin1–17	up in F	up in aL3
3.30	53549	Carnitine palmitoyl transferase (CPT)	up in F	nc
6.68	04080		M specific	down in aL3
G2.38	05677	Phosphate transporter	F specific	down in aL3
G2.38	08715		Male specific	up in aL3
G5.7	44862		Adult specific; down in F	

1 From Wang et al, 2010 ^[^31^]^.

2 Change in relative transcript reads between adult female (F) and male (M) libraries. Up, up regulated; down, down regulated.

3 Change in relative transcript reads between infective L3 and activated L3 (aL3) libraries.

Clustering the retrieved cDNAs yielded 22 transcript contigs. One r*Ac*-DAF-16 bound fragment was allowed to be linked to one or two transcript contigs. Sequence alignment between those transcripts and genome scaffolds revealed that r*Ac*-DAF-16 bound fragments resided in coding regions, introns, or 3′ untranslated regions (3′-UTR) (Table S3). However, not all the relative locations, especially the upstream ones, could be detected by this method due to incomplete annotation of the hookworm genome. Transcript sequences were searched against protein databases in Genbank and Gene Ontology Consortium for prediction of their function based on homology. The results classified r*Ac*-DAF-16 linked transcripts into two groups. The first contained transcripts homologous to known or hypothetical proteins, and displayed a variety of putative biological processes such as intra- and extracellular signaling (PKA, NLP-2, SRT-42), metabolism (CPT-II, ABC transporter class F, phosphate transporter), development (cuticlin), and transcription/translation (SNR-3); the second contained the transcripts without any identified homologs across available protein databases and therefore represent putative *A. caninum* specific genes.

The recent studies of Wang et al [31] generated 1.5 million *A. caninum* cDNAs from four developmental stages of *A. caninum*: infective L3 larva (L3), serum stimulated (activated) L3 larva (aL3), adult male (M), and adult female (F), covering approximately 93% of the *A. caninum* transcriptome. This dataset allowed construction of a stage-specific digital expression profile for many transcripts [31]. Examining the available expression pattern profiles indicated that five r*Ac*-DAF-16 linked transcripts (PKA, contig00807, contig25170, contig12656, and contig08715) were significantly up-regulated and three (contig40879, contig04080, and phosphate transporter) were significantly down-regulated by serum stimulation (table 2, Table S3), which is hypothesized to mimic the very early events of hookworm infections [1]. Three r*Ac*-DAF-16 linked transcripts (contig00807, contig25170, and NLP-2) are larval specific, and therefore turned off during development from L3 to adult. A major difference between parasitic hookworms and free-living *C. elegans* is that hookworms are dioecious, and the differential digital expression profile indicated that nine r*Ac*-DAF-16 linked transcripts are gender-specific (PKA, contig12656, contig40879, cuticlin1-17, CPT, contig04080, phosphate transporter, contig08715, and contig44862). Therefore, the DAF-16 linked transcripts identified here are involved in multiple biological processes during at least 4 hookworm developmental stages.

## Discussion

During infection, hookworm L3 resume developmental programs that had been arrested during the environmental stage, resulting in the successful establishment of a parasitic relationship with their host. Most of the molecular events associated with this “transition to parasitism” are still unknown. Dauer recovery of the free-living nematode *C. elegans* has long been used as a model for investigating the mechanisms of this transition to parasitism due to the biological similarities between the dauer stage and infective L3 of hookworms [1], [2], [3]. Specifically, the conserved insulin signaling pathway, which is required for *C. elegans* to exit the dauer stage in response to the improving environmental conditions, is also involved in the hookworm infection process [32], [33], [34]. Studies using inhibitors have shown that the ILS pathway is involved in hookworm larval activation, and the hookworm ortholog of the central transcription factor in this signaling pathway, DAF-16, has been identified and characterized [17], [35]. Furthermore, *Ac*-DAF-16 was transcriptionally active and interacted with hookworm 14-3-3 protein in a phosphorylation-status dependent way in cultured mammalian cell [17], [18].

DAF-16 is a FOXO transcription factor that is negatively regulated by ILS [4]. It functions in numerous biological processes, including metabolism, life span, stress responses, and dauer formation and exit, by mediating downstream gene expression in response to environmental and nutritional conditions [36]. Many of the downstream targets have been identified in *C. elegans*, and provide insights into the mechanism by which DAF-16 mediates multiple phenotypes [20], [21], [22], [23]. While many of these mechanisms are conserved in hookworms, the life cycles of parasitic nematodes differ significantly from that of *C. elegans*, and at least some of the DAF-16 outputs are likely to mediate processes specific to nematode parasitism. Therefore, fundamental mechanistic questions about hookworm infection can be addressed by the identification of DAF-16 binding sites and direct gene targets, and how those downstream effectors are coordinated in hookworm development.

To begin identifying *Ac*-DAF-16 target genes, we used an *in vitro* genomic selection technique to enrich for genomic fragments that were bound by the recombinant *Ac*-DAF-16 DBD. *In vitro* genomic selection with transcription factors differs from other affinity-based strategies because it uses the native genomic background, and therefore direct transcription factor targets are identified without influences from complex cellular environments [37]. The cyclical strategy selects binding sites with relatively high affinity and reduces indirect or nonspecific binding. In the present study, a group of genomic fragments with high affinity for r*Ac*-DAF-16 DBD were enriched, as indicated by higher-than-expected occurrence of some genomic fragments. Sequence analysis indicated that more than half of those fragments contained a reported consensus DBE or DBE-like element, and these elements were present in four of the five most represented DBD bound genomic fragments. The DBE or DBE-like elements occurred as single or multiple copies, and the element orientations and nucleotide spacers between the elements varied. As *C. elegans* DAF-16 and its mammalian homologs bind to this consensus DBE, the presence of the DBE in *Ac*-DAF-16 selected hookworm genome fragments suggests a conserved function for hookworm DAF-16. However, nearly half of the fragments we isolated did not contain a canonical DBE or DBE-like sequence, suggesting that the DAF-16 DBD bound to a previously unknown binding element. Further analysis of these fragments identified an over-represented 6-bp element, 5′- GAC/GAA/TG -3′. The *Ac*-DAF-16 DBD bound to amplicons containing this element, but not individual oligos, suggesting that the new element requires surrounding sequence for DBD binding. Our results do not rule out the presence of additional DAF-16 binding elements in the hookworm genome. It is not unusual that a single transcription factor has variable bind sites [38]. In any case, we have identified a new, previously unreported DAF-16 DBD binding element (GAC/GAA/TG) in hookworms, supporting a role for *Ac*-DAF-16 in multiple, perhaps novel, hookworm-specific biological processes.

The availability of the draft genome of *A. caninum* and comprehensive expression data enabled a detailed analysis of the DBD-bound genes. Twenty four of 25 r*Ac*-DAF-16 DBD selectively bound fragments were confirmed as *A. caninum*, and their proximal genomic regions were analyzed for coding regions. A total of 22 transcripts within a 6 kb range surrounding r*Ac*-DAF-16 DBD bound fragments were identified as *Ac*-DAF-16 primary target genes. The use of a 6 kb search range was based on *C. elegans* transcription factor binding site analyses [20], [34]. However, not all the genomic sequence hits in the present study returned transcript contigs within this range. Therefore, an extended search might be necessary to identify them, as *A. caninum* has a larger genome than *C. elegans* [39] and the *A. caninum* genome project is still underway.

Functions of the proteins encoded by the transcripts were predicted based on their homology to known or hypothetical proteins. This analysis suggested that DAF-16 is involved in a variety of biological processes. For example, PKA is a conserved serine/threonine kinase, activated by second messenger cAMP, and converts various extracellular signals into intracellular processes [40]. Serpentine receptors are G protein-coupled transmembrane receptors that play important roles in *C. elegans* chemoreception [41]. The NPL protein is critical for synaptic transmission between neurons [42], and membrane transporters are involved in transport of a wide variety of substrates across extra- and intracellular membranes [43], [44]. Cuticlins comprise the insoluble, high-order material in nematode cuticle and determine the developmental morphology and mobility of the worms [45]. Small nuclear riboproteins (SNR) are a part of RNA post-transcriptional modification machinery [46]. Among these genes, the serpentine receptor and ABC transporter were also reported in previous studies of DAF-16 targets in *C. elegans*, indicating that some pathways downstream of DAF-16 are conserved in free living and parasitic nematodes [20], [22], [23]. Additionally, some r*Ac*-DAF-16 DBD linked gene transcripts failed to match any homologs by exhaustive search of the available databases, and were defined as *A. caninum* specific. These are of particular interest, as they are absent from *C. elegans* and consequently might be involved in parasitism. Furthermore, several of these molecules could be envisioned functioning during the transition to parasitism based on homology and expression apttern.

Combining the transcriptomic and genome sequences revealed the relative location of *Ac*-DAF-16 binding sites to their linked genes in the *A. caninum* genome. However, this method is biased towards identification of *Ac*-DAF-16 binding sites located between exons due to incompleteness of hookworm genome annotation and the inability to identify 5′ ends and promoter sequences. Nonetheless, all 10 fragments that could be definitively linked to a gene had binding elements in introns or downstream sequences, suggesting that they may be regulated differently from genes with DBEs in the promoter. In *C. elegans*, DAF-16 target genes containing DBEs located downstream of the start codon were more likely to be negatively regulated by DAF-16 [47]. Characterizing intergenic *Ac*-DAF-16 binding sites will depend on further information about the genomic structure for the corresponding genes. The present study surprisingly indicates that *Ac*-DAF-16 binding sites, unlike *C. elegans* DAF-16 binding sites, reside at variable locations relative to the gene transcripts [23], suggesting that *Ac*-DAF-16 might regulate expression of genes with diverse functions and exert its action through different mechanisms.

In the absence of functional information for most of the identified r*Ac*-DAF-16 linked genes, evidence of differential expression is the most important source for prioritizing future investigations. Using the extensive cDNA dataset available for *A. caninum*, the expression profile for the gene transcripts have been quantitatively analyzed by comparing the frequency of EST occurrence in the different cDNA libraries. Examination of the expression patterns for the identified transcript contigs in the present study suggests that *Ac*-DAF-16 regulates gene expression in all hookworm developmental stages studied, including exit from developmentally arrested infective L3 stage, maturation to adults, and sexual differentiation in adults.

Using the affinity-based *in vitro* genomic selection procedure, we have shown for the first time that *Ac*-DAF-16 directly binds to response elements in the hookworm genome. The relative location of *Ac*-DAF-16 bound elements to the linked genes is variable, with an apparent bias towards downstream locations. The *Ac*-DAF-16 direct target candidates that were identified include both conserved and *A. caninum* specific genes, and will be subject to future functional investigations. With more comprehensive screening such as chromatin immunoprecipitation and improved *A. caninum* genome and transcriptome data, more *Ac*-DAF-16 downstream targets will be detected. Subsequent manipulation of these genes may lead to novel avenues for intervention in the hookworm life cycle.

## Materials and Methods

### *Ac-*DAF-16 DBD cloning, expression and purification

To clone the *Ac*-DAF-16 DBD, a fragment containing the C-terminally FLAG-tagged DBD (corresponding to amino acids 214-314) was amplified from a cDNA clone of *Ac*-DAF-16 isoform b, pCMV4-daf16 [17]. The specific forward primer (DAF16-DD-E-FLAG-FN: 5′–GATACCATGGGCAA TGCGTGGGGTAATCTC-3′, containing restriction site *Nco*I, underlined) and reverse primer (DAF16-DD-E-FLAG-RH: 5′–GATCAAGCTT *ATCGTCG TCATCCTTGTAGTC* *CAAGGTGGAGGCTCG AAC-3′*, containing restriction site *Hind*III, and the FLAG Tag, italics), were incubated with the template in a PCR. The cycling conditions were 2 min at 95°C; followed by 35 cycles of 1 min at 95°C, 1 min at 55°C, 1 min at 72°C and a final extension for 6 min at 72°C. The purified amplicon was digested with *Nco*I and *Hind*III, ligated into expression vector pET28a (Novagen) cut with the same restriction enzymes, and transformed into *Escherichia coli* DH5α competent cells. The expression construct contained both an in-frame FLAG tag and an in-frame hexahistidine (His) tag at C-terminus of the *Ac*-DAF-16 DBD, as confirmed by DNA sequencing (Nevada Genomics Center, Reno, NV).

The resulting plasmid DNA (pET28a-*Ac*-DAF-16 DBD/FLAG/His) was transformed into *E. coli* Rosetta (DE3) competent cells (Strategene), and expression was induced by addition of 1 mM isopropyl-β-D-thio-galacto-pyranoside (IPTG) to log-phase bacterial culture at 37°C for 4 hrs. An aliquot of *E. coli* Rosetta (DE3) cells were removed from the culture prior to induction to serve as a pre-induction control, and second aliquot from a culture grown under the same conditions except in the absence of IPTG served as an un-induced control. Induced bacterial cells and control bacterial cells were collected by centrifugation at 5000 rpm for 15 mins. After cell lysis by sonication in the presence of protease inhibitors (Pierce, Thermo Scientific, Rockford, IL), the expressed recombinant *Ac*-DAF-16 DBD (r*Ac*-DAF-16 DBD) was affinity-purified by Ni-NTA resin (Qiagen, Valencia, CA). Purified r*Ac*-DAF-16 DBD peptide was fractionated by SDS-PAGE eletrophoresis through 4–20% Tris-glycine pre-cast Novex gradient gels (Invitrogen, Carlsbad, CA), and examined by staining with Coomassie Brilliant Blue R250 (Sigma, St. Louis, MO) and Western Blot with anti-His (C-term) antibody (Invitrogen). The concentration of the expressed peptide was determined by the Micro BCA Protein Assay (Thermo Scientific).

### *Ac*-DAF-16 DBD Binding Assay

The DNA binding ability of r*Ac*-DAF-16 DBD was tested by streptavidin bead pull-down assay using double stranded DNA as described previously [17]. Briefly, 5′ end biotin-labeled forward oligonucleotides and their unlabeled complements for the DBE or predicted motif elements were synthesized (IDT, Coralville, IA). Forward and reverse oligonucleotides were annealed to form double-stranded (ds) DNA and 100 pmoles were used in the pull-down assays. To label selected genomic fragments, 5′ end biotin-labeled T7 primer was synthesized and used to amplify the fragment from a plasmid clone by PCR. The amplicons were purified and 2 pmoles were used in the pull-down assays. Biotin-labeled dsDNA was incubated with 200 ng of *Ac*-DAF-16 DBD in binding buffer (10 mM Tris pH 7.5, 50 mM KCl, 1 mM DTT, 2.5% glycerol, 5 mM MgCl_2_, 50 ng/ µL poly (dI •dC), 0.05% Nonidet P-40) at 4°C for 2 h. After incubation, 25 µl of 30% Streptavidin–Sepharose (GE Healthcare) slurry equilibrated with TNE_50_ buffer (10 mM Tris, 50 mM NaCl, 1 mM EDTA, pH 7.5)+0.1% Nonidet P-40 were added to the mixture and incubated for 2 h at 4°C. Beads were collected by centrifugation at 2500 *g* and washed twice with TNE_100_ buffer (10 mM Tris, 100 mM NaCl, 1 mM EDTA pH 7.5) +0.1% Nonidet P-40. The bound proteins were separated on a 4–20% SDS–PAGE and Western blotted with anti-FLAG antibody (Sigma).

### Hookworm genomic DNA preparation

The Baltimore strain of *A. caninum* (US National Parasite Collection #100655.00) was maintained in beagles as previously described [48]. The George Washington University Medical Center Institutional Animal Care and Use Committee approved this study (protocol #A147). Infective L3 larvae were recovered from coproculture by a modified Baermann technique and stored in buffer BU (50 mM Na_2_HPO_4_, 22 mM KH_2_PO_4_,70 mM NaCl, pH 6.8) [49] at room temperature until used.

Eighty thousand hookworm L3 were frozen in liquid nitrogen and ground to a fine powder using a pre-chilled mortar and pestle. Following physical disruption of the worms, hookworm genomic DNA was isolated using the Wizard SV Genomic DNA Purification System (Promega) according to the manufacture's instructions. Briefly, digestion solution master mix containing RNase A (75 µg/mL) and proteinase K (1.5 mg/mL) was added to the homogenate and the sample was incubated at 55°C overnight. The sample was then mixed with lysis buffer, applied to the minicolumn assembly, and eluted with buffer TE (10 mM Tris.HCl, 1 mM EDTA, pH 8.0). Fifteen micrograms of *A. caninum* genomic DNA were digested with 48 units of *Bfu*CI restriction endonuclease (NEB) overnight and stored at −20°C. The quality of DNA was examined by 0.8% agarose gel electrophoresis and the concentration was determined using a NanoDrop ND-1000 spectrophotometer.

### *In vitro* Genomic Selection

Thirty microliters of anti-FLAG M2 affinity matrix (Sigma) were rinsed with TBS buffer (10 mM Tris HCl, pH 7.4, 150 mM NaCl, 0.1 mM EDTA) three times. Two micrograms of purified r*Ac*-DAF-16 DBD/FLAG/His were immobilized on the prepared matrix by incubation for 2 hours at 4°C in 150 µL of TBS buffer. The matrix was then washed with TBS buffer containing 1 M NaCl, followed by two washes with the binding buffer (10 mM Tris at pH 7.5, 2.5% glycerol, 10 mM MgCl_2_, 50 mM KCl, 1 mM dithiothreitol (DTT), 0.05% NP-40). All washes were performed at 4°C for 5 min on a nutating mixer. Immobilized *Ac*-DAF-16 DBD was then incubated with 5 µg of *Bfu*CI digested *A. caninum* genomic DNA, in 150 µL of binding buffer containing 50 ng/ µL poly (dI•dC) for 30 min at room temperature on a nutating mixer. To control for non-specific binding and subsequent PCR enrichment of spurious genomic fragments, a control selection using only the anti-FLAG M2 affinity matrix was performed under identical conditions. Protein-DNA matrix complexes were washed with 150 µL binding buffer for 5 min on a nutating mixer at 4°C, followed by a wash with binding buffer containing 250 mM KCl and a third wash containing 500 mM KCl. Washing conditions were optimized with the consensus DBE, and 500 mM KCl was shown to be sufficient to remove most of the unspecific binding. *Ac*-DAF-16 DBD-bound DNA was eluted from the matrix with 250 µL of the binding buffer containing 1 M KCl (10 min with 150 µL followed by 15 min with 100 µL) on a nutating mixer. Two hundred and fifty microliters of TE (10 mM Tris HCl, pH 8.0, 1 mM EDTA) were then added to the eluate. The eluted DNA was phenol/chloroform-extracted and ethanol-precipitated in the presence of 5 µg of glycogen. The DNA pellet was re-dissolved in 12 µL H_2_O. The following oligonucleotides, which contain the *Not*I restriction site, were synthesized: forward *Not*I*Bfu*CI (5′-AAAAGGGGCGGCCGC-3′) and reverse *Not*I*Bfu*CI (5′-GATCGCGGCCGCC CCTTTT-3′), and annealed to form double-stranded (ds) *Not*I*Bfu*CI oligomer DNA for use in a ligation reaction. One pmole of ds *Not*I*Bfu*CI oligomer was ligated to 10 µL of the re-dissolved *Ac*-DAF-16 DBD bound DNA with T4 DNA ligase in 20 µL total volume. Two microliters of the ligation product were used as template in PCR reactions with *Not*I*Bfu*CI PCR primer (5′-AAAAGGGGCGGCCGCG ATC-3′). The cycling conditions were 4 min at 95°C, followed by 29 cycles of 1 min at 95°C, 1 min at 57°C, 2 min at 72°C and a final extension for 6 min at 72°C.

Amplified PCR products were purified with NucleoSpin Extract II kit (Clontech). Two micrograms of the DNA were used for subsequent rounds of binding experiments, as described above. After each round of binding and elution, PCR was performed for a total of 4 rounds. Purified PCR products from the last round were subcloned either into the *Not*I site of pBluescript KS+ vector (Stratagene) or directly into pGEM -T Easy vector (Promega) for sequencing and analysis.

### Motif analysis of hookworm DAF-16 bound genomic fragment

Constructs containing *Ac*-DAF-16 bound fragments from the *in vitro* genomic selection cloned in pBluescript KS+/pGEM-T were sequenced with vector specific primer T7. The sequence chromatograms were screened using sequence display software Chromas 2.33 (Technelysium Ltd.) to identify high quality regions, vector linker regions, and the low complexity regions. All subsequent analyses were based on the high-quality sequences with vector linker sequences removed.

Nucleotide sequences were searched against the draft *A. caninum* genome scaffold sequences [39] (Mitreva, unpublished) using BLAST to confirm the identity of the hookworm genomic fragments. If the overall percent identity of an alignment was above 95%, the genome scaffold was assumed to be a hit. The bound fragments that failed to hit any *A. caninum* genome scaffolds were further searched against nucleotide databases at GenBank (http://www.ncbi.nlm.nih.gov/) to detect possible contamination from other genomes. The scaffold hits were next analyzed for their coding potential by mapping the cDNAs to the genome sequence (see below).

Given the current knowledge of reported FOXO transcription factor binding sites, *Ac*-DAF-16 bound *A. caninum* genome fragments were first inspected for existence of the consensus DBE, 5′-TTG/ATTTAC-3′ (and the corresponding reverse compliment 5′- GTAAAC/TAA -3′) [19], or its reverse counterpart, 5′-CATTTA/GTT-3′ (and the corresponding reverse compliment 5′-AAT/CAAATG-3′). The motif discovery algorithm Gibbs Motif Sampler [50], [51] was applied to sequences lacking canonical binding sites to seek over-represented motifs as additional candidate *Ac*-DAF-16 response elements. The default eukaryotic parameters were used to run the Gibbs Motif Sampler program with a slight modification made to the target motif widths, with 6, 8, 10, 12, and 14 bp chosen based on the width range of *C. elegans* transcription factor binding sites in Open REGulatory ANNOtation database (ORegAnno) [52], [53]. Two other motif discovery algorithms, MEME [54] and Mdscan [55], were also attempted for de novo motif discovery; however, unlike the robust Gibbs Motif Sampler, either their performance in detecting the consensus DBE with a set of positive control sequences was poor or the parameter setting was not flexible. Therefore, only Gibbs Motif Sampler was used for the current data set. Returned motifs were ranked based on the occurrence rates in the bound genomic fragments. Sequence logos for identified motifs were generated using WebLogo [56]. All of the cDNA and genomic sequences are available at the Nematode.net FTP site (http://nematode.net/FTP/index.php) [57].

### Identification of potential *Ac*-DAF-16 regulated genes

*Ancylostoma caninum* genome scaffold sequences containing *Ac*-DAF-16 bound genomic fragments, stage specific *A. caninum* expressed sequence tag (EST) databases [58], and the recent *A. caninum* cADNA databases [31] were obtained from Nematode.net [57]. The available data sets cover 93% of the *A. caninum* transcriptome.

For each genome scaffold hit, the genomic template including the *Ac*-DAF-16 bound region and up to 3 kb extensions at both ends were extracted. Those templates were searched against EST databases/transcriptome databases using BLAST to identify corresponding transcripts. The cut-off for the overall percent identity of an alignment was 93% and a length of at least a 100 bp. Gene location was predicted with greater confidence if more ESTs were aligned. Gaps between two adjacent exon segments in an EST alignment were treated as possible introns and were confirmed by checking intron boundary sequences (GT/AG rule)[59]. The transcript contigs were then derived from those ESTs.

The identified transcripts were compared with existing protein sequences at GenBank for functional annotation, using a maximum E-value of 1×10^−10^ and a minimum of 50% similarity as cut-off. Their expression specificity across different developmental stages (L3, aL3, F and M) were characterized using a statistical approach defined by Audic [31], [60] Briefly, the cDNAs were organized into transcript contigs, and ESTs were grouped to the corresponding cDNA libraries derived from different developmental stages for each transcript contig. The frequencies of library specific cDNAs for each contig were recorded and analyzed using a modified Fisher's exact test with a significance of p<1 e-05. This allowed definition of the stage specificity of transcript expression with significance. Transcripts were also mapped to the three organizing principles of the Gene Ontology (GO) based on sequence similarity displayed using AmiGo (http://amigo.geneontology.org), and are available at Nematode.net (http://nematode.net).

## Supporting Information

Table S1 GenBank accession numbers and dbGSS identification numbers of *Ancylostoma caninum* genomic fragments isolated by genomic selection.
(0.02 MB XLS)

Table S2 Frequencies of DBE containing genome fragments recovered from immobilized r*Ac*-DAF-16 DBD genomic selection and control genomic selection.
(0.03 MB DOC)

Table S3 Identity and stage-specific relative transcript levels of *Ac*-DAF16-DBD-selected genes.
(0.02 MB XLS)
