# Supplementary material for: Identification of Hookworm DAF-16/FOXO Response Elements and Direct Gene Targets
Source: PLoS One. 2010 Aug 19;5(8):e12289. doi: 10.1371/journal.pone.0012289 (PMC2924398; doi:10.1371/journal.pone.0012289)
Supplement: Table S2 — Frequencies of DBE containing genome fragments recovered from immobilized rAc-DAF-16 DBD genomic selection and control genomic selection. (0.03 MB DOC) [file pone.0012289.s002.doc]

Table S2. Frequencies of DBE containing genome fragments recovered from immobilized r*Ac*-DAF-16 DBD genomic selection and control genomic selection

|  | **DAF-16 DBD selection** | **Control selection** |
| --- | --- | --- |
| **Total constructs sequenced** | 311 | 211 |
| **High quality Sequences** | 274 | 190 |
| **Unique genome fragments** | 24 ^a^ | 103 |
| **DBE containing genome fragments^b^ (%)** | 13 (54%) | 5 (5%) |
| **Predicted new motif (% of sequences)** | GACAAG (46%) | AGGAAGAG (36%) |

^a^ Not including sequences containing microsatellites.

^b^ Includes DBEs that differ by a single base from the canonical DBE (TTG/ATTTAC).
